# Left distal sciatic giant solitary myxoid neurofibroma: a case report & literature review

**DOI:** 10.3389/fsurg.2024.1417418

**Published:** 2024-08-08

**Authors:** Fatima az-Zahra’ Thawabtah, Mayar Idkedek, Hafez Nimer

**Affiliations:** ^1^ Medical Research Club, Faculty of Medicine, Al-Quds University, Jerusalem, Palestine; ^2^ Department of Neurosurgery, H-Clinic Hospital, Ramallah, Palestine

**Keywords:** neurofibroma, myxoid, giant, sciatic nerve, solitary

## Abstract

**Introduction:**

Neurofibroma, a rare benign tumor of the peripheral nervous system, can manifest anywhere along a nerve from the dorsal ganglion to its terminal branches. Myxoid neurofibroma can present as a solitary non-tender nodule and is often confirmed by positive immunohistochemical staining for S-100 protein. However, in 50% of cases, neurofibromas are associated with neurofibromatosis.

**Case presentation:**

We present a case of a 34-year-old male with mild pain in the posterior part of his left thigh, accompanied by a slowly-growing swelling particularly noticeable when flexing his knee. It had gradually increased in size over several months, which the patient observed as a decrease in the degree of knee extension. Initial biopsy indicated schwannoma with no evidence of malignancy. Four years later, the swelling increased in size and necessitated resection surgery, revealing an irregular giant tumor measuring 8 *6 *4.5 cm, adherent to adjacent structures, including the femur, muscles, popliteal artery and vein, and a branch of the sciatic nerve. Pathological analysis reclassified the diagnosis to low-grade myxoid neurofibroma. Follow-up MRI three months later showed gross total resection without residual or recurrence of the tumor.

**Discussion:**

Solitary neurofibromas are often small in size, ranging from 1 to 2 cm in the greatest dimension. Alternatively, tumors that occur as a part of genetic neurofibromatosis tend to be multiple and often grow to large sizes. In our case, the patient didn't have neurofibromatosis as he didn't meet its diagnostic criteria despite having a giant tumor measuring approximately 8*6*4.5 cm. To our knowledge, this is the first report of giant myxoid solitary neurofibroma of the thigh apart from neurofibromatosis. Thus, this type of tumor should be considered in the differential diagnosis of tumors at this location.

## 1 Introduction

The peripheral nervous system encompasses nerves and ganglia emerging from the brainstem or spinal cord, extending throughout the body to provide autonomic and somatic innervation (1). Its wide anatomical distribution and histological complexity contribute to the diversity of morphological and biological types of tumors associated with it (2).

Peripheral nerve sheath tumors (PNST) are neoplasms that arise from the cell components of the peripheral nerve sheath. They can be categorized as benign, benign yet potentially locally aggressive, and malignant. There are several types of PNST including neurofibroma, schwannoma, and malignant peripheral nerve sheath tumor (2, 3).

Neurofibroma is a benign tumor of the peripheral nervous system, that can occur anywhere along a nerve from the dorsal ganglion to its terminal branches (4, 5). It is composed of a variable mixture of fibroblasts, Schwann cells, perineural-like cells, and cells with intermediate features between them. It is also considered a rare tumor and, along with schwannoma, one of the most common forms of peripheral nerve sheath tumors (4).

PNST can develop sporadically or can be linked to familial history and genetic predisposition, the latter contributing to genetic syndromes; such as neurofibromatosis type 1, neurofibromatosis type 2, schwannomatosis, and the Carney complex (6). Neurofibromas may present sporadically, or as part of neurofibromatosis (7). Solitary neurofibroma might manifest in one of the following forms: cutaneous lipomatous, collagenous, epithelioid, granular, pigmented, dendritic cell, or myxoid neurofibromas (8). Neurofibromatosis is considered as a genetic disorder that causes multiple tumors on nerve tissues involving the spinal cord, peripheral nerves, and brain (9).

Myxoid neurofibroma, a relatively uncommon variant of solitary neurofibroma, is characterized by bland spindle cells lacking cytologic atypia and mitosis within a myxoid matrix. It is rarely encountered in cytology specimens and consists of benign spindle cell tumors originating from perineural cells (10, 11). Positive immunohistochemical staining for S-100 protein can confirm its diagnosis. It often presents as a solitary non-tender nodule, though it can be numerous. This tumor has a higher incidence in young adults. The usual first-line treatment is total excision of the tumor, but it may recur after incomplete initial excision (12).

Here, we report a case of giant myxoid neurofibroma that was located distally in the posterior aspect of the left thigh, originating from the sciatic nerve, measuring 8*6*4.5 cm, without being associated with neurofibromatosis.

## 2 Case description

### 2.1 Patient information

A 34-year-old male patient was in his usual state of health until 4 years prior to his presentation when he developed mild pain on the posterior part of his left thigh. Subsequently, he noticed a non-tender swelling in the same region, firm in consistency, and particularly noticeable when flexing his knee. The swelling had gradually increased in size over several months, which the patient observed as a decrease in the degree of knee extension. The patient reported no additional symptoms; such as numbness, weight loss, or fatigability. His past medical and surgical histories were unremarkable, and he had no relevant family history. After seeking medical advice, an open biopsy was performed aiming at evaluating the mass. The first histopathological report indicated evidence of schwannoma without any signs of malignancy.

### 2.2 Clinical findings

However, four years later, the tumor had progressively enlarged, causing pain and swelling at the same site and limitation of knee flexion. The patient returned for further evaluation. No obvious clinical signs of neurofibromatosis were observed, and genetic testing for NF1 and NF2 genes showed negative results (13).

### 2.3 Diagnostic assessment

**Figure 1:**
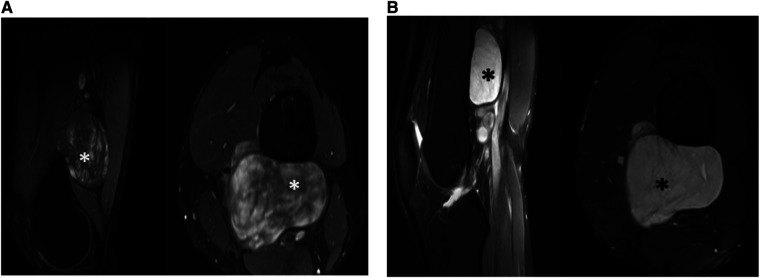
Tumor observed by pre-operative (**A**) MRI T1W with contrast sagittal and axial (**B**) MRI PDFS sagittal and axial.

Left thigh magnetic resonance imaging (MRI) revealed multiple, variable-sized, well-circumscribed soft tissue lesions located in the posterior compartment of the lower third of the thigh, deep to the extensor muscles. These lesions appeared to be closely related to adjacent neurovascular bundles. The MRI exhibited an inhomogeneous high signal on T2-weighted image/Short tau inversion recovery (T2WI/STIR) with faint internal septations and intense heterogeneous enhancement on post-contrast sequences (Figure 1).

### 2.4 Therapeutic intervention

**Figure 2:**
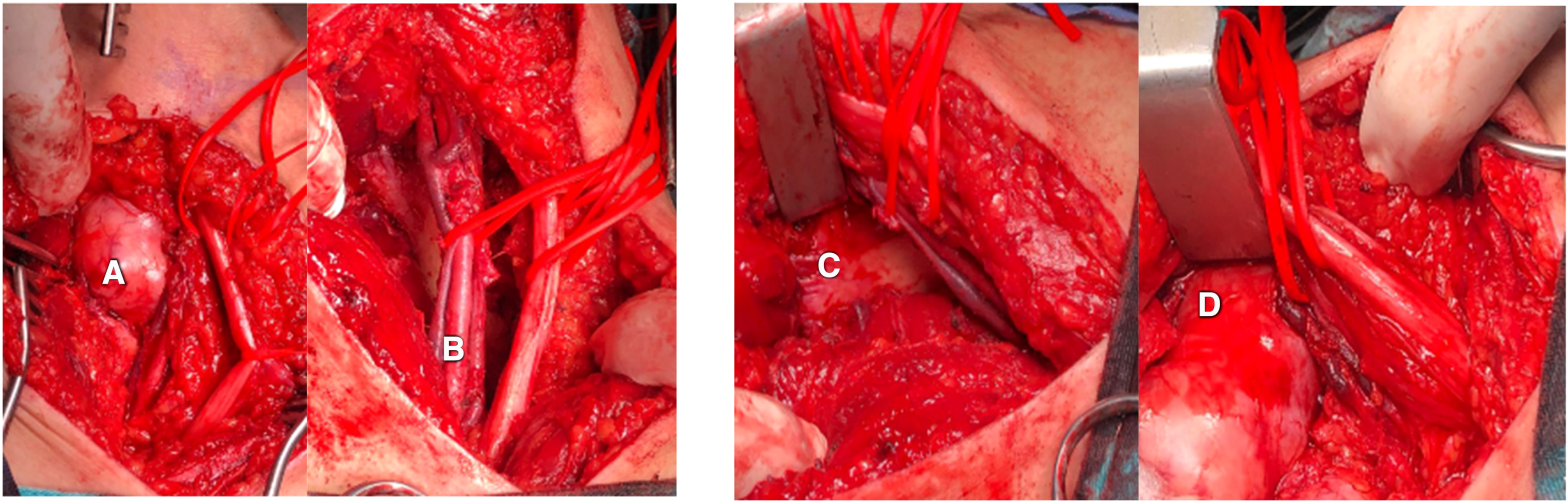
(**A**) The tumor was attached to surrounding structures including (**B**) the neurovascular bundle composed of the popliteal artery and vein, and sciatic nerve, and (**C**) the femur bone. (**D**) Tumor attachment to the femur bone.

**Figure 3:**
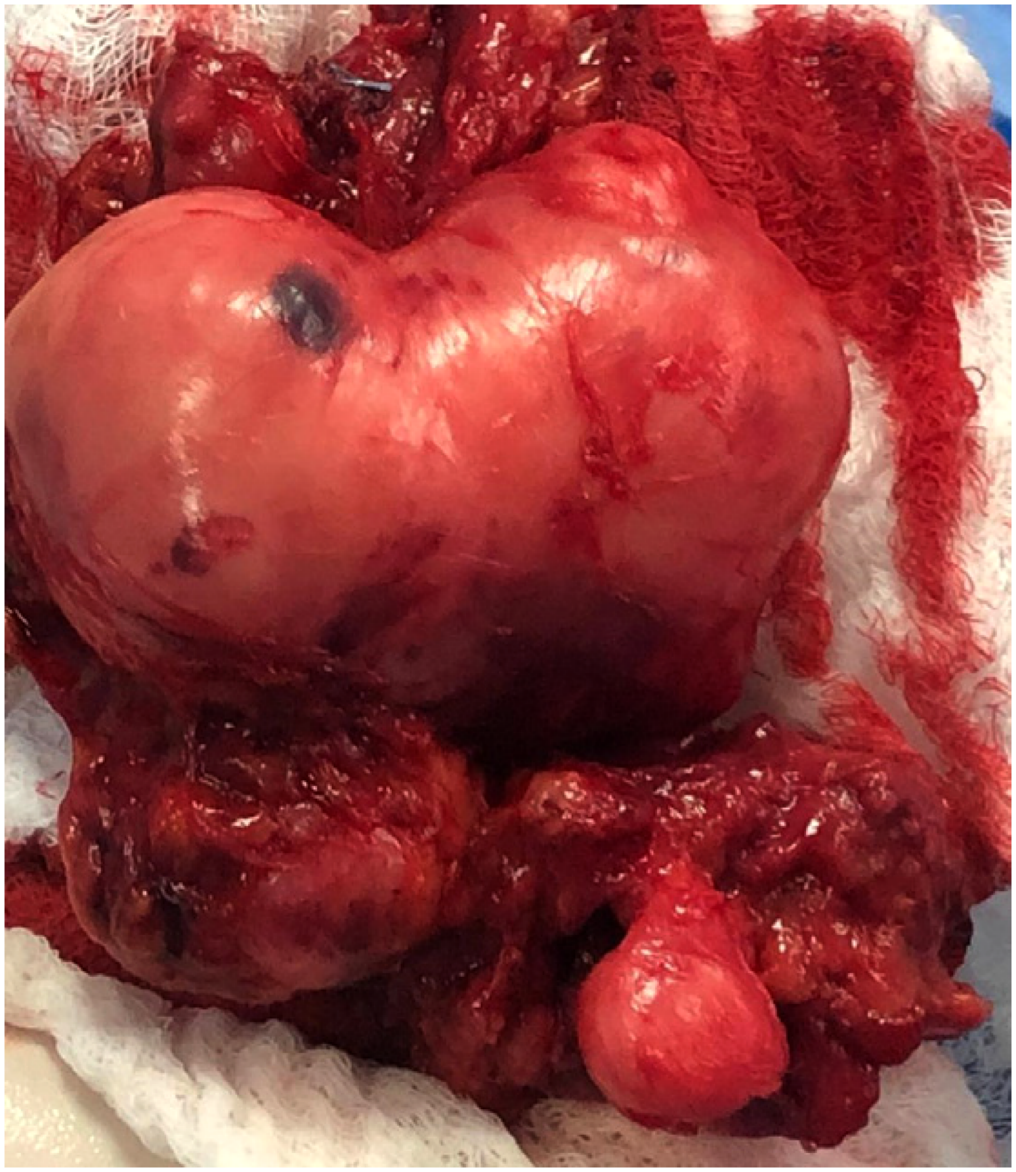
(**A**) Huge resected tumor with an irregular shape, grayish tissue covered by fat and skeletal muscle, and a measurement of 8 × 6 × 4.5 cm.

Consequently, the patient underwent resection surgery to dissect the mass. The surgery was performed under general anesthesia with the patient in a prone position. The left lower limb was attached with needles for continuous electromyography, and an incision was made along the midline of the left posterior thigh, exposing the fascia and subcutaneous layer. The tumor resection began proximally and continued until reaching the sciatic nerve and its branches. The resection revealed strong adhesion of the tumor to the femur, posterior thigh muscles, popliteal artery and vein, and sciatic nerve branches, as depicted in Figure 2. Due to this strong adhesion, a sciatic nerve branch and the tumor's neurovascular bundle had to be removed. Hemostasis was carefully achieved, and the excision successfully freed the neurovascular bundle. The neuromonitoring showed no alterations, and a vacuum drain was inserted into the resection cavity. The subcutaneous tissue was approximated and sutured using 2/0 interrupted vicryl, the skin was closed with clips, and the dressing was applied. Grossly, a large irregular tumor with daughter cysts was removed, presenting as grayish tissue covered by fat and skeletal muscle, measuring 8*6*4.5 cm (Figure 3).

### 2.5 Follow-up and outcomes

Two days postoperatively, the patient's condition had improved; he was able to move his leg, and his left foot power was 5/5 with intact sensation. There were no focal neurological deficits. The wound was dry, and the drain was removed. He was discharged on the second day with a prescription for Etoricoxib at a daily dosage of 90 mg for 14 days, Tramadol three times daily for 10 days, and Cefpodoxime 200 mg twice daily for 10 days.

The new histopathological findings have led to a revision of the initial diagnosis from schwannoma to low-grade myxoid neurofibroma. The pathology report described the mass as irregular, surrounded by a thin capsule, with a white and mucoid cut surface, and the resection margins were unremarkable.

**Figure 4:**
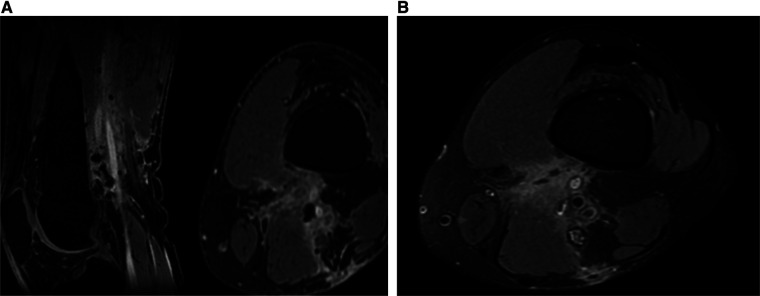
No enhancing mass lesions in post-operative (**A**) MRI T1W contrast sagittal and axial (**B**) MRI PDFS axial

Two weeks later, the patient underwent a follow-up visit at the outpatient clinic. During the visit, he appeared well, and the surgical wound exhibited cleanliness with no signs of infection. Three months later, the patient returned for another follow-up visit, reporting mild popliteal paresthesia. Neurological examination was intact. MRI was conducted, revealing a thickened distal part of the sciatic nerve, no enhancing mass lesions or fluid collection, and postoperative changes with enhancing soft tissue and hypointense foci (surgical material) (Figure 4). In conclusion, the MRI indicated a gross total resection of the tumor without residual or recurrence of the tumor. Table 1 describes a timeline table that details the patient's complaints and procedures occurring over a period of five years.

### 2.6 Timeline

**Table 1:** A timeline table that describes the patient's complaints and procedures over a period of five years.

Year	Event
2018	The patient presented with a left posterior distal thigh swelling
2018	Excisional biopsy for the mass was done and histopathological reports showed schwannoma
2022	The tumor had grown into a larger size, total resection surgery was done and new histopathological reports changed the diagnosis to Myxoid neurofibroma
2022	Three months postoperatively, the patient presented with mild popliteal paresthesia. MRI was done and showed gross total resection of the tumor without residual or postoperative changes.

## 3 Discussion

Neurofibroma is a rare benign tumor of the peripheral nervous system that constitutes a common form of peripheral nerve sheath tumors. It is capable of occurring at almost any point within nerve distribution, with a predilection for the head and trunk (4, 5).

Neurofibroma affects both genders equally, displaying no association with race or ethnicity. Onset can occur at any age, but isolated masses are most frequently encountered in individuals aged 20–40 years, as was the case with our patient at the age of 34 (13).

Solitary neurofibromas typically manifest as small lesions, often measuring 1–2 cm in the greatest dimension. Conversely, those associated with genetic neurofibromatosis are usually multiple and can attain larger sizes (14). Remarkably, our case presented a dissected tumor measuring 8*6*4.5 cm, without a genetic association, providing valuable educational insights. Myxoid neurofibromas are generally solitary and our case mirrored this pattern (12). Local recurrence of neurofibroma is uncommon after complete mass excision. Our patient experienced tumor progressive enlargement over years after the initial biopsy (15).

The anatomical distribution of solitary neurofibromas is diverse, including locations; such as the retroperitoneal space, spine, nose, cheek mucosa, and others (16). Myxoid solitary neurofibromas have been reported in sites like the palm, forearm, soft palate, floor of the mouth, and thumb. To our knowledge, our case represents the first reported instance of a giant myxoid solitary neurofibroma in the thigh, underscoring the importance of considering this tumor type in the differential diagnosis for thigh tumors.

Presentations of neurofibroma can vary, with many cases being asymptomatic. Deep lesions may present with pain, numbness, paresthesias, and mass effect (17).

While neurofibroma usually occurs sporadically, approximately 10% of cases exhibit familial history (18). In about 50% of cases, neurofibroma is associated with neurofibromatosis type 1 syndrome (19). There was no significant family history in our case, and neurofibroma occurred independently of neurofibromatosis syndromes.

Schwannomas and neurofibromas are both types of neurogenic tumors originating from Schwann cells, but they exhibit distinct differences in gross examination and histological characteristics. Schwannomas typically appear as well-encapsulated tumors with possible cystic changes, whereas neurofibromas are non-encapsulated and tend to be more invasive into surrounding soft tissues. Histologically, schwannomas exhibit specific cell patterns; such as Antoni Type A and Type B patterns, along with Verocay bodies, characterized by elongated, whorling patterns of palisading nuclei. In contrast, neurofibromas lack these structures and typically consist of spindle cells within a loose collagenous matrix, along with fibroblasts, mast cells, and axons. Confirmation of diagnosis is achieved through pathology examination (20).

Surgical resection of neurofibroma poses challenges because it may lack a clear capsule, increasing the risk of fascicle injury (21). Employing a consistent microsurgical technique supported by a surgical microscope, stimulating forceps, and appropriate microsurgical instrumentation is crucial to minimize postoperative neurological damage (22) In our case, the tumor originated from a sciatic nerve branch in the distal thigh, requiring careful resection, yet postoperative results demonstrated no nerve injury, with the patient experiencing only a slight sensory deficit in the popliteal area.

## Data Availability

The datasets presented in this article are not readily available because this is a case report, so no database was generated. Requests to access the datasets should be directed to fatimathawabtah731@gmail.com.
